# Lower urinary tract symptoms that predict microscopic pyuria

**DOI:** 10.1007/s00192-017-3472-7

**Published:** 2017-10-02

**Authors:** Rajvinder Khasriya, William Barcella, Maria De Iorio, Sheela Swamy, Kiren Gill, Anthony Kupelian, James Malone-Lee

**Affiliations:** 1 Division of Medicine, Centre for Nephrology, Division of Medicine, UCL Medical School, Hornsey Central Neighbourhood Health Centre, 151, Park Road, London, N8 8JD UK; 2 Department of Statistical Science, University College London, London, UK

**Keywords:** Urinary tract infection, Pyuria, Microscopy, Lower urinary tract symptoms, Validated symptom score

## Abstract

**Introduction and hypothesis:**

Urinary dipsticks and culture analyses of a mid-stream urine specimen (MSU) at 10^5^ cfu ml^−1^ of a known urinary pathogen are considered the gold standard investigations for diagnosing urinary tract infection (UTI). However, the reliability of these tests has been much criticised and they may mislead. It is now widely accepted that pyuria (≥1 WBC μl^−1^) detected by microscopy of a fresh unspun, unstained specimen of urine is the best biological indicator of UTI available. We aimed to scrutinise the greater potential of symptoms analysis in detecting pyuria and UTI.

**Methods:**

Lower urinary tract symptom (LUTS) descriptions were collected from patients with chronic lower urinary tract symptoms referred to a tertiary referral unit. The symptoms informed a 39-question inventory, grouped into storage, voiding, stress incontinence and pain symptoms. All questions sought a binary yes or no response. A bespoke software package was developed to collect the data. The study was powered to a sample of at least 1,990 patients, with sufficient power to analyse 39 symptoms in a linear model with an effect size of Cohen’s f^2^ = 0.02, type 1 error probability = 0.05; and power (1-β); 95% where β is the probability of type 2 error). The inventory was administered to 2,050 female patients between August 2004 and November 2011. The data were collated and the following properties assessed: internal consistency, test–retest reliability, inter-observer reliability, internal responsiveness, external responsiveness, construct validity analysis and a comparison with the International Consultation on Incontinence Modular Questionnaire for female lower urinary tract symptoms (ICIQ-FLUTS). The dependent variable used as a surrogate marker of UTI was microscopic pyuria. An MSU sample was sent for routine culture.

**Results:**

The symptoms proved reliable predictors of microscopic pyuria. In particular, voiding symptoms correlated well with microscopic pyuria (χ^2^ = 88, df = 1, *p* < 0.001). The symptom inventory has significant psychometric characteristics as below: test–retest reliability: Cronbach’s alpha was 0.981; inter-observer reliability, Cronbach’s alpha was 0.995, internal responsiveness F = 221, *p* < 0.001, external responsiveness F = 359, df = 5, *p* < 0.001. The correlation coefficients for the domains of the ICIQ-FLUTS were around *R* = 0.5, *p* < 0.001.

**Conclusion:**

This symptoms score performed well on the standard, psychometric validation. The score changed in response to treatment and in a direction appropriate to the changes in microscopic pyuria. It correlated with measures of quality of life. It would seem to make a good candidate for monitoring treatment progress in ordinary clinical practice.

## Introduction

Urinalysis by dipstick and/or midstream urine culture (MSU) is the first investigation of the patient presenting with lower urinary tract symptoms (LUTS). Negative results are usually assumed to exclude infection and diagnoses such as overactive bladder (OAB) depend on negative tests. The MSU is still considered the gold standard diagnostic test for UTI. It relies on isolating 10^5^ cfu ml^−1^ of a known urinary pathogen, using aerobic culture with a* Enterobacteriaceae*-selective media. This was first described by Kass [1] after studying patients with chills, fever, flank pain and dysuria. Kass never claimed to define a threshold for use with “cystitis”, i.e. frequency/dysuria. However, 10^5^ cfu ml^−1^ has been widely adopted. The dipstick has been validated against a standard of 10^5^ cfu ml^−1^ of a known urinary pathogen.

In fact, these urinalysis methods are not sensitive and are incapable of excluding UTI. Effort is being made to find improved urinalysis techniques to detect UTI [2], but currently the best performing method is microscopy of a fresh unspun, unstained, clean-catch specimen of urine in a counting chamber to enumerate the white cells [3].

Our past reliance on dipstick analysis and culture to diagnose UTI has now been rejected in the literature [4], we should therefore re-examine our understanding of the symptoms and signs involved in LUTS [5].

The analysis of symptoms presents different challenges to the measurement of change over time, when assessing treatment outcomes. Well-known validated scores of LUTS such as the International Consultation on Incontinence Questionnaire (ICIQ), [6] the Incontinence Quality of Life Questionnaire (ICI-QOL) [7] and Female Lower Urinary Tract Symptoms (F-LUTS) [8] are used to assess the impact of incontinence. They have performed well in measuring the effects of treatments in clinical trials. Their utility in single, cross-sectional analyses is less assured because they use adjectival scaling such as “bothersomeness”. People differ greatly in their semantic interpretation of adjectives. In longitudinal studies, the potential error from this variance can be avoided by the normalisation of within subject changes. This option is available to neither cross-sectional studies, nor to data collected at the time of diagnostic assessment. Additionally, scores such as the ICIQ were validated in patients after UTI had been excluded on the evidence of urine culture. Our interest lies in the symptoms associated with reliable indicators of UTI.

We aimed to design a symptom analysis method, free of adjectival scaling, covering the spectrum of LUTS. We wished to avoid selecting symptoms based on our assumptions. The symptom inventory was constructed from sets collected from patients who, unconstrained, described their experience of disease. We chose to use “Yes/No” responses to enquiry to avoid adjectival qualification. We have reported this approach in developing descriptive measures in several different circumstances. We have found that it is possible to achieve valid scaling by counting the different circumstances that aggravate a patient’s symptoms.

The study aim was to build a symptoms inventory from the patient’s descriptions of their experiences. Once assured that this could be applied by using dichotomised responses, we planned to validate the psychometric properties of the questionnaire. We then planned to examine the relationship to the pathological condition by regression analysis on microscopic pyuria, which is currently our best marker of UTI [3, 9]. We also arranged to test the responsive properties of the questionnaire during treatment.

## Materials and methods

Ethics committee approval was obtained from the East London and the City Research Ethics Committee. All patient data were anonymized after collection.

### The evolution of the symptom set

The first task was to identify symptoms that described, as thoroughly as possible, the experience of lower urinary tract infections (LUTS) from the patients’ perspective in patients referred with chronic LUTS to a tertiary referral centre. A computer programme was created that enabled a clinician to record symptoms while the patients narrated them. The software functioned to accumulate lists that were easy to add to. A core set of ubiquitous symptoms rapidly evolved, with rarer and idiosyncratic experiences being added as more patients participated. At the end of their narrative, patients were asked if they were confident that they had described everything. This method was deployed in our ordinary clinical service between 1991 and 1999.

In 1999, the data were analysed and we extracted all symptoms that occurred at least as frequently as dysuria (≥2% of respondents), because the latter is an archetypal symptom of cystitis. These questions were then grouped into the four main categories: storage and voiding symptoms, stress urinary incontinence (SUI) and pain. These questions were organised into an inventory that was used to elicit symptoms from patients by direct questioning, recording a “yes” or “no” response. The patients were always asked to supplement the information with additional material applicable to their experience. In 2004, the data were analysed again and a final inventory was constructed from those symptoms described by ≥2% of respondents. These 39 questions are listed in Table 1.

**Table 1:** Symptom inventory in the order in which the questions are asked

Storage symptoms	Stress symptoms	Voiding symptoms	Pain symptoms
1. Urgency	12. Cough sneeze incontinence	20. Hesitancy	27. Suprapubic pain
2. Urge incontinence	13. Exercise incontinence	21. Reduced stream	28. Filling bladder pain
3. Latchkey urgency	14. Laughing incontinence	22. Intermittent stream	29. Voiding bladder pain
4. Latchkey urgency incontinence	15. Passive incontinence	23. Straining to void	30. Post-void bladder pain
5. Waking urgency	16. Bending incontinence	24. Terminal	31. Pain relieved by voiding
6. Waking urge incontinence	17. Incontinence	25. Post-void dribbling	32. Partially voiding relief
7. Running water urgency	18. Lifting incontinence	26. Double voiding	33. No voiding relief
8. Running water urge incontinence	19. Pre-cough preparation		34. Loin pain
9. Cold urgency			35. Iliac fossa pain
10. Anxiety urgency			36. Pain radiation to genitals
11. Premenstrual aggravation			37. Pain radiation to legs
			38. Dysuria
			39. Urethral pain

Questions 29 to 32 were introduced in this format in 2004

These two preparatory phases sampled males and females so as to avoid a gender bias in the structure of the final symptom inventory.

### Validation of the symptom set

The 39-question set was written into a new database and from 2004 these questions were included in the assessment of all adult (≥18 years) patients with untreated LUTS, presenting to a secondary care facility attached to urology and urogynaecology departments. The observers presented the question set by using a script (see Appendix Table 4). The reported 24-h urinary frequency and incontinence were recorded. A group of normal control subjects also contributed data.

At the time of assessment, a clean-catch midstream urine sample was collected as described below. From this an immediately fresh, unspun, unstained specimen was examined in a haemocytometer as described below. An aliquot was despatched for routine culture, as described below.

The first part of the data analysis involved psychometric validation according to the methods described below.

#### Reliability

1. Internal consistency, which measured the within-group agreement, when the questions were collected into the following categories: storage symptoms, voiding symptoms, SUI symptoms and pain symptoms. This was assessed by calculation of Cronbach’s alpha coefficient.
2. Test–retest reliability, which measured the consistency of the symptom inventory when repeated in the same patient. This was done by allowing each patient to complete the symptom set twice within 2 days, so that the symptoms were unlikely to have changed. We used Cronbach’s alpha coefficient for the paired data.
3. Inter-observer reliability, which measured the consistency of the symptom inventory when performed by different observers of the same patient. This was carried out by two observers questioning the same patient independently during the same clinic attendance. We used Cronbach’s alpha coefficient for the paired data.

Thus, reliability was assessed according to the ICIQ guidelines [6].

#### Responsiveness

1. Internal responsiveness, the ability of the symptom set to detect change, was measured by comparing the symptoms over four successive follow-up visits during treatment. The patients were treated for LUTS symptoms, using bladder retraining, antimuscarinic drugs for OAB symptoms and antibiotics if microscopic pyuria was identified. The response was assessed using mixed model linear regression.
2. External responsiveness was analysed first by comparing of the symptom score with the patient’s grading of the treatment response on the scale: “worse”, “no change”, “mild improvement”, “moderate improvement”, “marked improvement”, and second by comparing the inventory with 24-h frequency and 24-h incontinence. The response was assessed using mixed model linear regression.
3. Construct validity analysis formed the crux of this study. We explored the inferences that could be drawn from symptom occurrence in relation to the probability of significant microscopic pyuria, the latter being our best indicator of UTI. To analyse this, we used mixed model linear regression.

### Comparison with FLUTS

We collected data from 135 patients who completed an International Consultation on Incontinence Modular Questionnaire for female lower urinary tract symptoms (ICIQ-FLUTS) [8] and compared the FLUTS subgroup scores with the number of symptoms detected by our inventory grouped according to the four categories: urgency, stress, voiding and pain. We used Spearman’s correlation coefficient.

### Urinalysis methods

The MSU samples were obtained by the midstream clean-catch method described elsewhere [10]. The microscopic leucocyte count was achieved as follows: a fresh aliquot of urine was split into two and examined by microscopy. 1 μl of urine was loaded into a clean Neubauer haemocytometer counting chamber [11] and the preparation examined by light microscopy (magnification, ×200). The white cells were counted per 1 μl. These data were collected by specially trained doctors and nurses. A routine MSU culture was performed as follows: an aliquot of all urine specimens collected was sent to the Whittington Hospital NHS Trust microbiology laboratory, as is routinely done, for culture using standard methods [10] The result was taken as positive if at least 10^5^ colony-forming units (cfu) ml^−1^ of a known pathogen were present, after 18–24 h of culture.

### Statistics

We used a mixed model linear regression analysis to scrutinise the log_10_ WBC as the response variable. This method is designed for repeated measures and copes with missing data cells. We had to address the occurrence of an excess of zero WBC counts and achieved this by using the glmmADMB procedure in R to construct a zero-inflated negative binomial regression model to accommodate over-dispersion of the data; the regression model was specified for the mean of the negative binomial. The zero WBC counts were expressed as very small numbers for the log_10_ transformation. The symptoms were treated as individual independent variables (39) questions.

To accommodate an interpretation, commensurate with common clinical practice, the patients were additionally grouped according to their pyuria counts: zero, pyuria 1–9 or pyuria ≥10, and then the symptoms were plotted against these groups (see Fig. 4) We did this because of the widespread use of pyuria ≥10 WBC μl^−1^ as the threshold for “significant” pyuria [12], although results that incriminate pyuria between 1 and 9 have been published [2].

To compare the voiding and pain symptoms among the three categories of pyuria, we used ordinal regression with the category as the dependent variable and the number of voiding symptoms and pain symptoms as covariates.

We used a two-tailed non-parametric test with an effect size (d) = 0.5, which means that an effect in either direction would be interpreted. The criterion for significance (probability of alpha error) was set at 0.050 and a power of 80% (1-beta error of probability = 0.8). With the proposed sample size of 170 for the two groups (43 controls and 127 patients), the study had a power of 80% to yield a statistically significant result with an effect size d = 0.5.

**Fig. 1:**
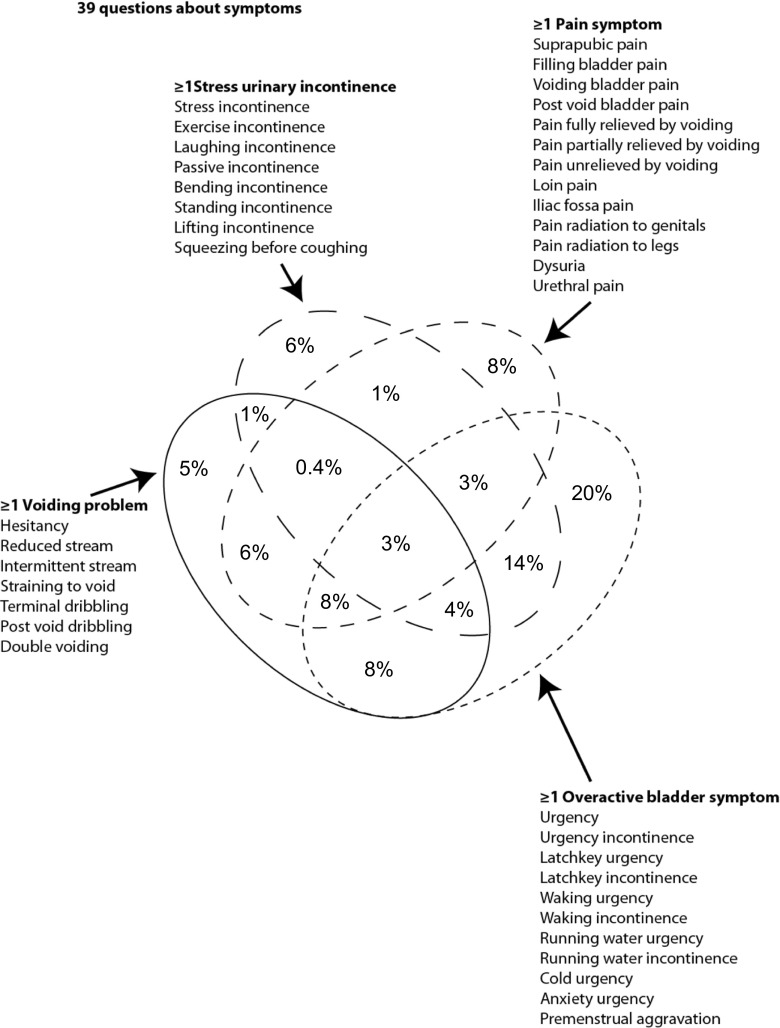
Four-way Venn diagram of symptom overlap

Cohen’s d effect size = 0.5 (Fig. 1) was selected, as this is the smallest effect that it would be important to detect, in the sense that any smaller effect would not be of clinical or substantive significance. It was also assumed that this effect size is reasonable, in the sense that an effect of this magnitude could be anticipated in this field of research. Cohen’s d is defined as the mean difference expressed in standard deviations. d = difference between means ÷ pooled standard deviations of the two groups.

The sample of at least 1,990 patients had sufficient power to analyse 39 predictor symptoms. Missing data were recorded as null fields.

## Results

The first data set was collected between January 1991 and July 1999 and analysed when the software was re-written to accommodate the millennium bug threat. It contained data from 2,446 patients; 2,019 (82%) female and 427 (18%) male with a mean age of 53 (SD = 19). During 7,467 consultations, they provided 65,535 data entries. The first question set, made up of each symptom being described by ≥2% of respondents, consisted of those depicted in Table 1 apart from questions 29 to 33.

The second data set, collected between August 1999 and July 2004, was analysed once we had validated our method for counting pyuria. There were data on 2,109 patients; 1,836 (87%) female and 273 (13%) male with a mean age of 52 (SD = 30). There were 7,046 consultations that provided 54,159 data entries. This analysis resulted in modification of the description of bladder pain, which was expanded into a four-question set: bladder pain or discomfort on filling; filling bladder pain relieved by voiding; filling bladder pain partially relieved by voiding; (filling bladder pain unrelieved by voiding (questions 29 to 33 in Table 1). We did this because these symptom qualifications, referring to voiding, were described by ≥2% of respondents (Table 1).

During the study phase, the LUTS symptom inventory was administered to 2,050 female patients presenting between August 2004 and November 2011. Their mean age 52 (SD = 17) and the average number of symptoms was 10 (SD = 5.9; median = 9), with a mean duration of symptoms of 6 years (SD = 6). The considerable overlap between the symptom groups is illustrated in the four-way Venn diagram of Fig. 1.

A total of 1,305 patients (64%) had no pyuria at presentation, 356 patients (17%) had pyuria between 1 and 9 WBC μl^−1^ and 389 (19%) had pyuria of ≥10 WBC μl^−1^. Two hundred and forty-five patients (12%) demonstrated positive MSU cultures.

### Reliability

#### Internal consistency

Cronbach’s alpha for urgency was 0.88 with 11 items, for pain it was 0.861 with 13 items, for stress incontinence 0.884 with 8 items and for voiding 0.882 with 7 items. When the symptoms were counted within each group, Cronbach’s alpha was 0.35 for the counts of urgency symptoms, stress incontinence symptoms, voiding symptoms and pain symptoms, confirming that the symptoms between groups measured different things.

The theoretical value of alpha varies from zero to 1. Higher values of alpha are more desirable. A reliability of 0.70 or higher is often required as a consensus before an instrument is used. 0.80–0.9 is good and >0.9 is excellent.

#### Test–retest reliability

Ten patients participated in the test–retest analysis. Cronbach’s alpha was 0.981 with an inter-item correlation coefficient of 0.974.

#### Inter-observer reliability

Ten patients participated in the inter-observer reliability analysis. Cronbach’s alpha was 0.995 with an inter-item correlation coefficient of 0.991.

### Responsiveness

#### Internal responsiveness

**Fig. 2:**
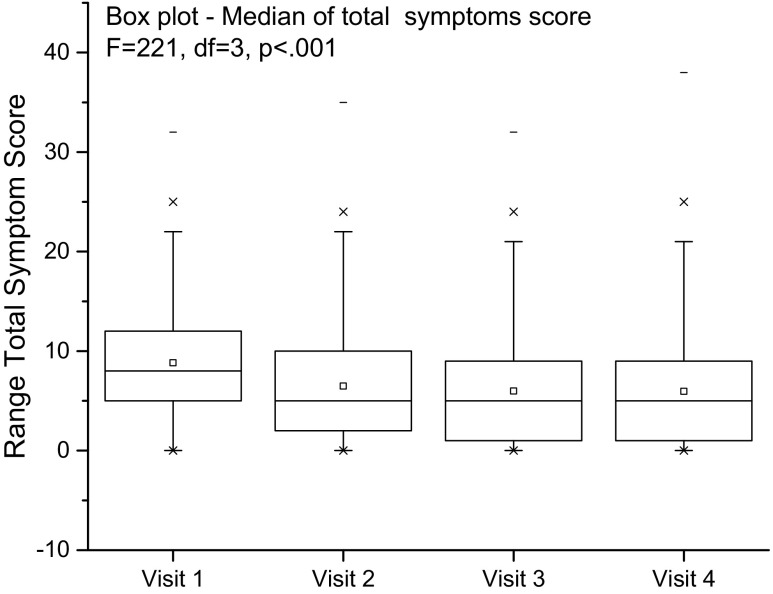
Internal responsiveness

A total of 2,050 patients provided data on follow-up over 4 visits (24 weeks); the patients were treated with a combination of antimuscarinics and antibiotics. Analysis showed a significant reduction in the total number of symptoms F = 221, *p* < 0.001. The tables from the mixed model analysis are shown in the Appendix (Fig. 2)

#### External responsiveness

We examined external responsiveness by comparing the number of symptoms as the dependent variable using the following independent variables: the patients’ assessment of overall response; 24-h frequency; 24-h incontinence.

**Fig. 3:**
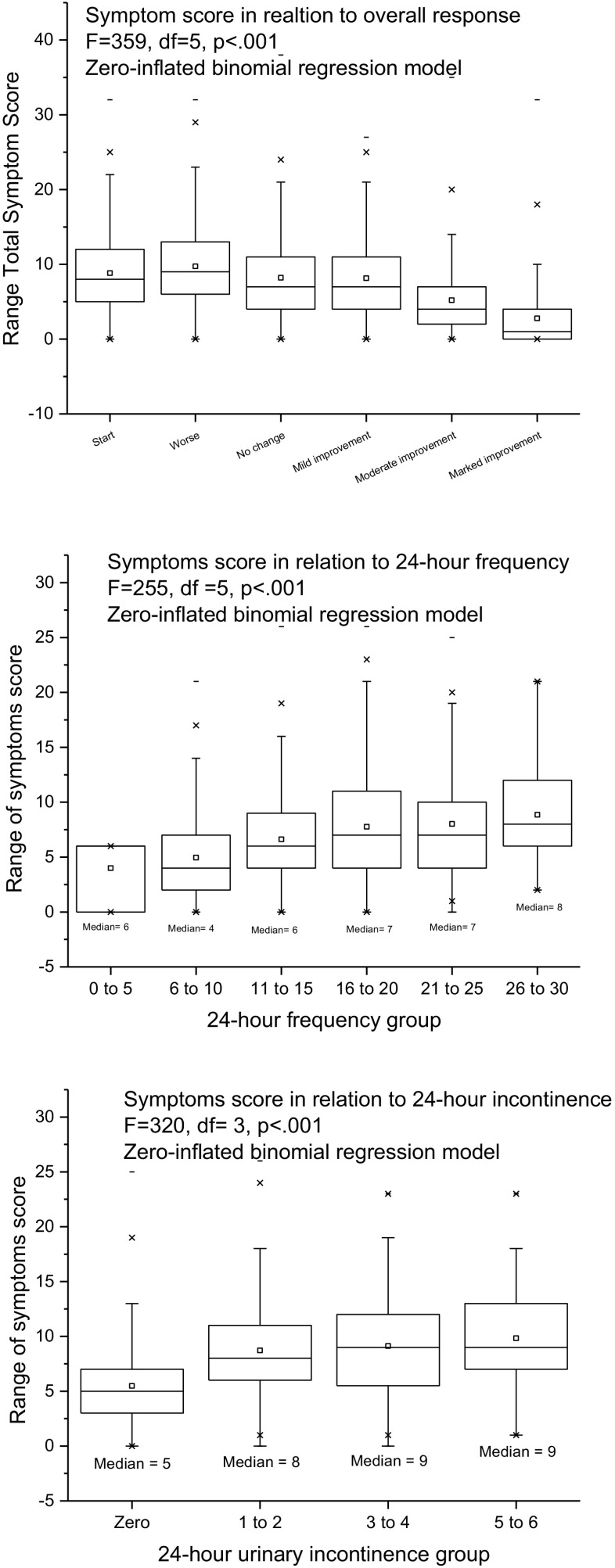
External responsiveness

The results were as follows. Overall response: F = 359, df = 5, *p* < 0.001; 24-h frequency: F = 255, *p* < 0.001; 24-h incontinence: F = 320, *p* < 0.001. Figure 3 plots the number of symptoms against the patients’ overall assessment of response, the 24-h urinary frequency, and urinary incontinence.

#### Construct validity

The zero-inflated negative binomial regression model parameter estimates and statistics, with pyuria (WBC μl^−1^) as the dependent variable; repeated measures identified by visit number; and with the independent covariates being age, 25-frequency and 24-h incontinence and the 39 symptoms (No = 0 Yes = 1) are shown in Table 2. Age, 24-h frequency, 24-h incontinence, urge incontinence, latchkey incontinence, bladder pain post-void, dysuria and reduced stream explained a significant proportion of the variance in log_10_ pyuria; exercise incontinence, passive incontinence and pre-cough guarding were negative explanatory variables.

**Table 2:** Zero-inflated negative binomial regression model parameter estimates and statistics

Coefficients	Estimate of “B”	Standard error	z value	*p* value	Significance (*p* value)
Intercept “C”	−0.07561	0.22852	−0.33	0.74075	
**Age**	**0.0292**	**0.0034**	**8.59**	**< 2e-16**	**<0.001**
**24-h frequency**	**−0.04465**	**0.01216**	**−3.67**	**0.00024**	**<0.001**
**24-h incontinence**	**0.10157**	**0.04891**	**2.08**	**0.03784**	**<0.05**
Urgency	**0.00945**	**0.15417**	**0.06**	**0.95113**	
**Urgency incontinence**	**−0.50046**	**0.16189**	**−3.09**	**0.00199**	**<0.001**
Latchkey urgency	**0.07889**	**0.1533**	**0.51**	**0.60682**	
**Latchkey incontinence**	**0.51879**	**0.1881**	**2.76**	**0.00581**	**<0.001**
Waking urgency	0.07302	0.14919	0.49	0.62454	
Waking urgency incontinence	0.34714	0.17846	1.95	0.05175	
Running water urgency	−0.19246	0.15407	−1.25	0.2116	
Running water urgency incontinence	−0.04143	0.20421	−0.2	0.83922	
Cold urgency	0.14011	0.1301	1.08	0.2815	
Anxiety urgency	0.06066	0.13452	0.45	0.65202	
Perimenstrual symptom aggravation	−0.02327	0.21281	−0.11	0.91291	
Suprapubic pain	0.13497	0.14545	0.93	0.35344	
Filling bladder pain	0.30853	0.31115	0.99	0.3214	
Voiding bladder pain	0.25012	0.17	1.47	0.14121	
**Post-void bladder pain**	**0.45377**	**0.1505**	**3.02**	**0.00257**	**<0.001**
Pain relieved by voiding	−0.42165	0.32854	−1.28	0.19935	
Pain partially relieved by voiding	−0.5755	0.32128	−1.79	0.07325	
Pain unrelieved by voiding	−0.27706	0.37857	−0.73	0.46425	
Loin pain	0.25804	0.14409	1.79	0.07332	
Iliac fossa pain	0.1135	0.14823	0.77	0.44384	
Pain radiation to genitals	−0.18812	0.15214	−1.24	0.21627	
Pain radiation to legs	−0.15892	0.17227	−0.92	0.35626	
**Dysuria**	**0.78097**	**0.15377**	**5.08**	**3.80E-07**	**<0.001**
Urethral pain	−0.00573	0.161	−0.04	0.9716	
Stress incontinence	0.18834	0.15313	1.23	0.21872	
**Exercise incontinence**	**−0.58106**	**0.20903**	**−2.78**	**0.00544**	**<0.001**
Laughing incontinence	0.08031	0.21176	0.38	0.70449	
**Passive incontinence**	**−0.43011**	**0.2182**	**−1.97**	**0.0487**	**<0.05**
Bending incontinence	−0.19502	0.24875	−0.78	0.43304	
Standing incontinence	0.29879	0.24378	1.23	0.22033	
Lifting incontinence	−0.04617	0.22139	−0.21	0.83479	
**Preparing before coughing**	**−0.86663**	**0.22553**	**−3.84**	**0.00012**	**<0.001**
Hesitancy	0.20904	0.14249	1.47	0.14236	
**Reduced stream**	**0.51777**	**0.14893**	**3.48**	**0.00051**	**<0.001**
Intermittent stream	0.24887	0.14587	1.71	0.08799	
Terminal dribbling	0.20226	0.14053	1.44	0.15008	
Double voiding	0.22458	0.14037	1.6	0.10961	
Postmicturition dribbling	0.04447	0.15421	0.29	0.77305	
Straining to void	0.04685	0.15315	0.31	0.75968	

The bold entries are highly significant

Negative binomial was preferred to Poisson likelihood to accommodate overdispersion of the data

The regression model was specified for the mean of the negative binomial and an inflation parameter was estimated to account for the out-of-pattern number of zeros

Regression (WBC ul^−1^ = B_i_x_i_ + B_i+1_ x_i+1_ + ……. B_j_x_j_ + C + error)

### The clinical implications

#### Comparison with FLUTS

The correlation between the FLUTS subgroups and the grouped symptoms from our inventory are shown in Table 3. The coefficients range from 0.36 to 0.57 and it is interesting that these were greatest for the voiding symptoms.

**Table 3:** Correlation between female lower urinary tract symptoms (FLUTS) subgroups and grouped symptoms. Correlation matrix from comparison with International Consultation on Incontinence Modular Questionnaire (ICIQ)

		Urgency symptom count	Stress incontinence symptom count	Voiding symptom count	Pain symptom count
ICIQ-LUTS-QOL; symptom score (Pearson correlation R)		0.449	0.434	0.449	0.346
ICIQ-LUTS-QOL; bother score (Pearson correlation R)		0.464	0.408	0.444	0.352
ICIQ-FLUTS; symptom score (Pearson correlation R)		0.487	0.419	0.543	0.365
ICIQ-FLUTS; bother score (Pearson correlation R)		0.528	0.481	0.586	0.392

All of these coefficients were statistically significant at *p* < 0.001

#### Symptom implications

**Fig. 4:**
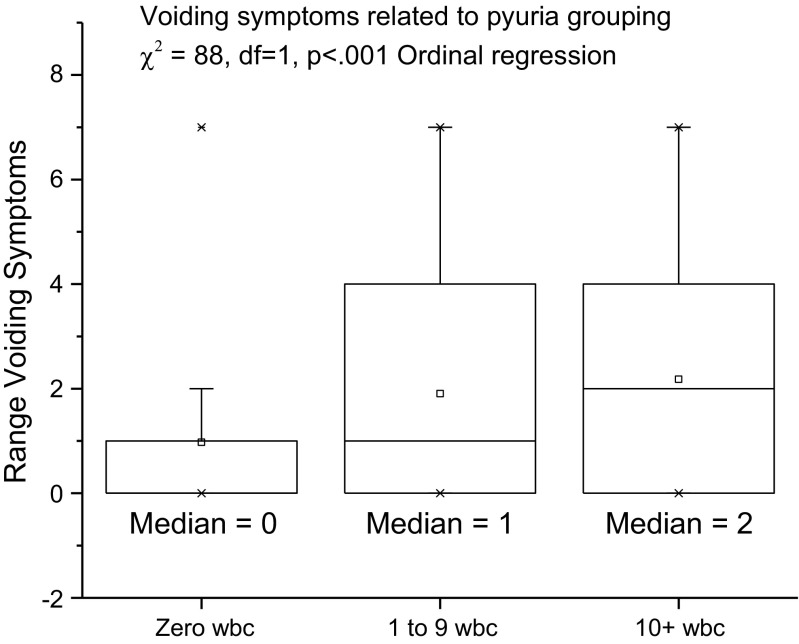
Voiding symptoms related to the pyuria group

**Fig. 5:**
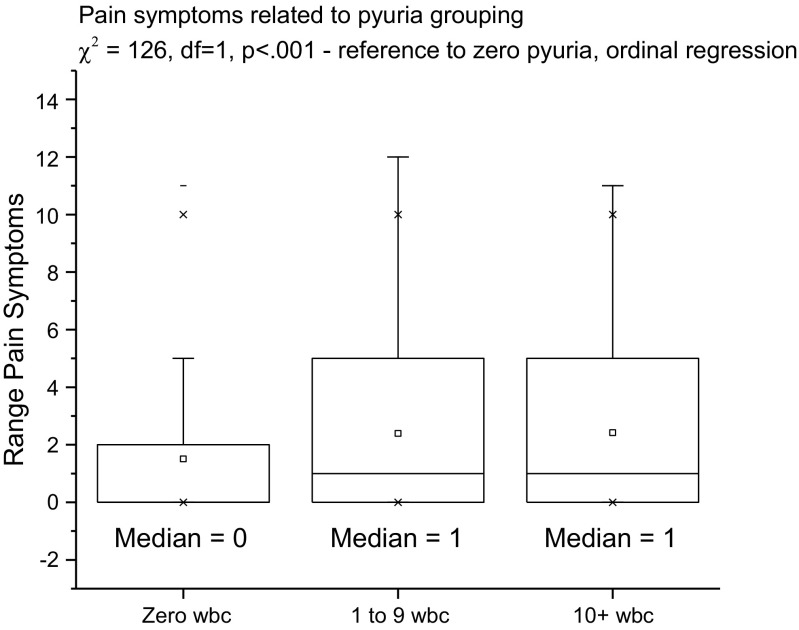
Pain symptoms related to the pyuria group

The core aim was to examine the relationship between the symptoms and microscopic pyuria as the best marker of UTI. The MSU cultures, only 12% positive, were not suitable for such analysis. An important finding was the prominence of voiding symptoms as indicators of pyuria. In particular, these seemed to be most discerning in the milder expressions of the disease. Figure 4 plots the voiding symptoms count (covariate) against the pyuria groups (dependent ordinal variable; zero, 1 to 9, ≥ 10) in patients not describing pain (χ^2^ = 88, df = 1, *p* < 0.001). The voiding symptoms count discriminated among all three categories. The pain symptom count only discriminated between zero pyuria and any pyuria (χ^2^ = 148, df = 1, *p* < 0.001; Fig. 5).

## Discussion

A most important finding from this study has been the discovery that in women, voiding symptoms play an important part in the complex associated with pyuria, pain features but is only evident for the higher levels of pyuria; it would seem that voiding symptoms are more discriminating.

There are many symptom scores reported in the literature that measure the experience of lower urinary tract disease, designed and validated by others. We would not have invested the effort required to develop this inventory without good reason. There is a growing realisation that the methods used to screen for urine infection in clinical practice are far from accurate and that their sensitivities do not justify the given status as arbitrators of the presence or absence of infection. We have recently reported that in patients with chronic LUTS, the dipstick test is at best 59% sensitive [3] and others have shown a diverse urinary microbiome in patients with incontinence and urgency when routine urine cultures are negative [9, 13]. In truth, we do not have reliable methods for achieving such decisive diagnoses. We must therefore rely on the symptoms and signs, which may be no bad thing. We must take a history, examine the patient and not delegate the diagnostic decision to a single test.

The questionnaire that is described in this paper was designed to measure the symptomatic manifestations of pyuria. We could not validate against proven UTI because there is no contemporary microbiological method capable of furnishing the necessary data with sufficient accuracy. Microscopic pyuria, measured by immediate microscopy of a fresh, unstained and unspun specimen of urine in a haemocytometer is the best marker of urinary infection that we have, despite its surrogacy [2].

The 39 questions that make up the questionnaire had their origin in the free texts collected when patients were asked to describe their condition in their own words. Many of the early years of this project were devoted to obtaining these data and fashioning them into an inventory requiring dichotomized responses. Fidelity to the original source data was maintained throughout so that the questionnaire was not altered to accommodate data from investigations or diagnostic categorization. The correlates show that the patients’ own descriptions of their states concur with the pathophysiology.

The symptoms score proved a good predictor of microscopic pyuria and monitor of disease progression. The dependent variable, pyuria, is the best surrogate marker of UTI available. The score correlated with measures of quality of life. Voiding symptoms were proved indicators of mild disease with pain indicating more severe inflammatory responses.

## Appendix

**Table 4:** The question inventory in the order in which the questions are asked

Storage symptoms		Stress symptoms		Voiding symptoms		Pain symptoms	
1. Urgency	Do you have to hurry to pass urine because you might wet yourself?	12. Cough sneeze incontinence	Do you leak on coughing or sneezing?	20. Hesitancy	Are you slow to start passing urine?	27. Suprapubic pain	Do you feel bladder pain over the pubic area?
2. Urge incontinence	Do you hurry to pass urine but not make it in time?	13. Exercise incontinence	Do you leak on exercise?	21. Reduced stream	Is the urinary stream reduced?	28. Filling bladder pain	Does the bladder pain worsen as the bladder fills?
3. Latchkey urgency	Do you find that you have to rush to pass urine when you put a key in the front door?	14. Laughing incontinence	Do you leak on laughing?	22. Intermittent stream	Does the stream stop and start?	29. Voiding bladder pain	Do you get bladder pain during voiding?
4. Latchkey urge incontinence	If you have to rush to pass urine when you put a key in the front door, do you ever leak with this?	15. Passive Incontinence	Do you leak for no good reason?	23. Straining to void	Do you have to strain to pass urine?	30. Post-void bladder pain	Do you get bladder pain after voiding?
5. Waking urgency	Do you have to hurry to pass urine because you might wet yourself when you wake up in the morning?	16. Bending incontinence	Do you leak on bending?	24. Terminal dribbling	Does the stream dribble at the end?	31. Pain relieved by voiding	Is bladder pain fully relieved by voiding?
6. Waking urge incontinence	If you have to hurry to pass urine when you wake up in the morning, do you ever wet yourself?	17. Standing incontinence	Do you leak on standing from sitting?	25. Postvoid dribbling	Does it dribble after you have finished?	32. Partialvoiding relief	Is bladder pain partially relieved by voiding?
7. Running water urgency	Do you have to hurry to pass urine because you might wet yourself on hearing running water?	18. Lifting incontinence	Do you leak on lifting anything?	26. Double voiding	Do you pass urine, leave the toilet, and then have to go back again?	33. No voiding relief	Is bladder pain partially unrelieved byvoiding?
8. Running water urge incontinence	If you have to hurry to pass urine on hearing running water, do you ever wet yourself?	19. Pre-cough preparation	Do you get ready to avoid leaking when you are about to cough?			34. Loin pain	Do you get flank pain in the kidney area?
9. Cold urgency	Do you have to hurry to pass urine because you might wet yourself on exposure to cold?					35. Iliac fossa pain	Do you get pain to the right or left of the low abdomen?
10. Anxiety urgency	Do you have to hurry to pass urine because you might wet yourself on when you are worried or anxious?					36. Pain radiation to genitals	Do you get pain radiating into the vagina?
11. Premenstrual aggravation	Do you have to hurry to pass urine because you might wet yourself, more around the time of your period?					37. Pain radiation to legs	Do you get pain radiating down your legs?
						38. Dysuria	Does it burn when you pass urine?
						39. Urethral pain	Do you experience pain in the urethra (the tube through which the urine passes?

## Abbreviations

F-LUTS: Female lower urinary tract symptoms
ICIQ: International Consultation on Incontinence Questionnaire
LUTS: Lower urinary tract symptoms
MSU: Mid-stream urine
OAB: Overactive bladder
QOL: Quality of life
UTI: Urinary tract infection
